# African manatee (*Trichechus senegalensis*) habitat suitability at Lake Ossa, Cameroon, using trophic state models and predictions of submerged aquatic vegetation

**DOI:** 10.1002/ece3.8202

**Published:** 2021-10-07

**Authors:** Aristide K. Takoukam, Dylan G. E. Gomes, Mark V. Hoyer, Lucy W. Keith‐Diagne, Robert K. Bonde, Ruth Francis‐Floyd

**Affiliations:** ^1^ African Marine Mammal Conservation Organization Edea Cameroon; ^2^ Department of Large Animal Clinical Sciences College of Veterinary Medicine University of Florida Gainesville Florida USA; ^3^ Cooperative Institute for Marine Resources Studies Hatfield Marine Science Center Oregon State University Newport Oregon USA; ^4^ UF/IFAS School of Forest Resources and Conservation Fisheries and Aquatic Sciences Florida LAKEWATCH Gainesville Florida USA; ^5^ African Aquatic Conservation Fund Joal Senegal; ^6^ Clearwater Marine Aquarium Research Institute Clearwater Florida USA

**Keywords:** bathymetry, conservation, endangered species, eutrophication, limnology, marine mammal, species distribution modeling

## Abstract

The present study aims at investigating the past and current trophic status of Lake Ossa and evaluating its potential impact on African manatee health. Lake Ossa is known as a refuge for the threatened African manatees in Cameroon. Little information exists on the water quality and health of the ecosystem as reflected by its chemical and biological characteristics. Aquatic biotic and abiotic parameters including water clarity, nitrogen, phosphorous, and chlorophyll concentrations were measured monthly during four months at each of 18 water sampling stations evenly distributed across the lake. These parameters were then compared with historical values obtained from the literature to examine the dynamic trophic state of Lake Ossa. Results indicate that Lake Ossa's trophic state parameters doubled in only three decades (from 1985 to 2016), moving from a mesotrophic to a eutrophic state. The decreasing nutrient gradient moving from the mouth of the lake (in the south) to the north indicates that the flow of the adjacent Sanaga River is the primary source of nutrient input. Further analysis suggests that the poor transparency of the lake is not associated with chlorophyll concentrations but rather with the suspended sediments brought‐in by the Sanaga River. Consequently, our model demonstrated that despite nutrient enrichment, less than 5% of the lake bottom surface sustained submerged aquatic vegetation. Thus, shoreline emergent vegetation is the primary food available for the local manatee population. During the dry season, water recedes drastically and disconnects from the dominant shoreline emergent vegetation, decreasing accessibility for manatees. The current study revealed major environmental concerns (eutrophication and sedimentation) that may negatively impact habitat quality for manatees. The information from the results will be key for the development of the management plan of the lake and its manatee population. Efficient land use and water management across the entire watershed may be necessary to mitigate such issues.

## Graphical abstract

Little information exists on the water quality and health of the aquatic ecosystem within protected areas in Cameroon. We monitor water quality in Lake Ossa, a refuge for the threatened African manatee, and find considerable eutrophication and poor water transparency. These results suggest that this manatee refuge may currently be of poor habitat quality and is in need of management intervention.

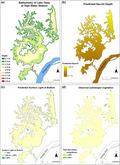

## 1 INTRODUCTION

During the past two centuries, the level of anthropogenic input of nutrients to the Earth's surface waters has increased as a result of the industrial revolution and population growth (Galloway et al., 2004; Lee et al., 2016). The large increase in the rate of land clearing, domestic animal production, agro‐industry, urbanization, dam construction, and other human activities has contributed to increased nutrient enrichment (eutrophication) of fresh and marine water systems, thus altering the natural hydrological cycles (Smith et al., 1999; Vitousek et al., 1997). Such increases can lead to uncontrolled proliferation of algal biomass, resulting in hypoxia and increasing light attenuation in the water column, thus limiting the growth of submerged plants. This can change the trophic state of bodies of water, such as lakes, and lead to devastating consequences for organisms that rely on submerged plants. In Florida, for example, the nutrient enrichment of Lake Okeechobee was suspected to cause the harmful algal bloom events that have led to the death of hundreds of Florida manatees (Broadwater et al., 2018).

The African manatee (*Trichechus senegalensis*) is a threatened aquatic mammal, classified as Vulnerable on the International Union for Conservation of Nature (IUCN) Red List (Keith‐Diagne, 2015). African manatees are found across 21 African countries in various habitat types, including coastal marine water, brackish estuaries and lagoons, rivers, and tributaries and lakes that connect to the sea. Manatee habitat requirements are thought to include, but are not limited to, access to warm, freshwater at least 2 m deep, food plant availability, access to cover, and adequate space (Reep & Bonde, 2006; USFWS, 1999).

During the low‐water season in Africa, water recedes away from the shoreline vegetation of major rivers. During these times, manatees tend to travel downstream to the estuaries or into adjacent lakes where they have access to deeper water (Powell, 1996). During high‐water seasons, the water levels increase and inundate vegetation and forest along the shoreline. Manatees travel to these flooded areas to consume the accessible fresh vegetation (Powell, 1996). Thus, freshwater lakes serve as an important resource for the African manatee, especially during low‐water periods. In Cameroon, which has been designated a top conservation priority (Rinnan & Jetz, 2020), African manatees are often sighted in Lake Ossa, which is part of a reserve established to provide a refuge for the African manatee. However, it is unclear how suitable the habitat at Lake Ossa is for African manatees, as the amount of edible vegetation, and water depth and quality were previously unstudied.

Manatees are mostly herbivorous and likely rely heavily on macrophytes as a food source. Florida manatees can eat an equivalent of 10–15% of their body weight of plants daily (Reep & Bonde, 2006). Manatees also use floating macrophyte beds to hide when the risk of detection by fishers is high (pers. obs. ATK). Thus, the aquatic plant productivity of Lake Ossa appears to be a major limnological component that determines the carrying capacity and the spatiotemporal abundance of manatees in a water system. Rooted macrophytes (as opposed to unrooted, free‐floating plants) mainly acquire nitrogen and phosphorous from sediments on the lake bottom substrate, which in turn primarily depends on the geology of the lake watershed (Canfield & Hoyer, 1988). In Lake Ossa, nutrients are mostly provided by runoff from the lake catchment basin or by the drainage of the Sanaga River that discharges highly turbid water into the lake (Wirrmann & Elouga, 1998). The sedimentation rate of Lake Ossa is relatively high, varying from 45 cm to 192 cm per 1000 years (Giresse et al., 2005). Therefore, rooted plants in Lake Ossa have large stores of phosphorous and nitrogen, and their growth is probably not limited by nutrient availability but by the amount of light reaching the surface of their leaves, especially given the extreme turbidity.

Light availability at water bottom can be a limiting factor for macrophyte photosynthesis owing to the high attenuation of irradiance (flux of light penetration per unit area) through the water column. Previous studies have validated this with an empirical model that demonstrates a positive linear relationship between water transparency and the maximum depth of plant colonization (MDC) (Caffrey et al., 2007; Canfield et al., 1985). Chlorophyll concentration (indicating algal productivity), dissolved organic matter, and suspended sediments can independently influence the transparency of water (Canfield & Hodgson, 1983; Carlson, 1977; Vant & Davies‐Colley, 1984). Chlorophyll concentration, in turn, can be influenced by nutrient concentration, especially phosphorus (Canfield, 1983). Therefore, it appears that a chain of relationship models can be established between nutrients, chlorophyll concentration, and water clarity. Water clarity, in turn, can be used to predict MDC and therefore submerged vegetation coverage.

Understanding the biological, chemical, and physical components of Lake Ossa is essential for understanding how African manatees utilize their habitat. The objectives of this study were as follows: (a) to assess the current trophic state of Lake Ossa, (b) to examine an empirical chain of trophic state models in Lake Ossa, and (c) to determine the relationship between the current trophic state of the lake and the abundance of submerged vegetation. By assessing the trophic state and establishing the bathymetry of Lake Ossa, we can determine potential implications for submerged aquatic vegetation and on habitat use by the threatened African manatee.

## 2 METHODS

### 2.1 Study area description

The Lake Ossa complex is located at 13 km from Edea, Cameroon, between the 3°45′N and 3°52′N latitude, and 9°45′E and 10°4′E longitude at approximately 300 m elevation (Wirrmann & Elouga, 1998). The water surface is estimated to be 4000 ha, which represents about 90% of the Lake Ossa Wildlife Reserve, created in 1968. The reserve was established to provide a refuge for the protection of the African manatee.

**FIGURE 1:**
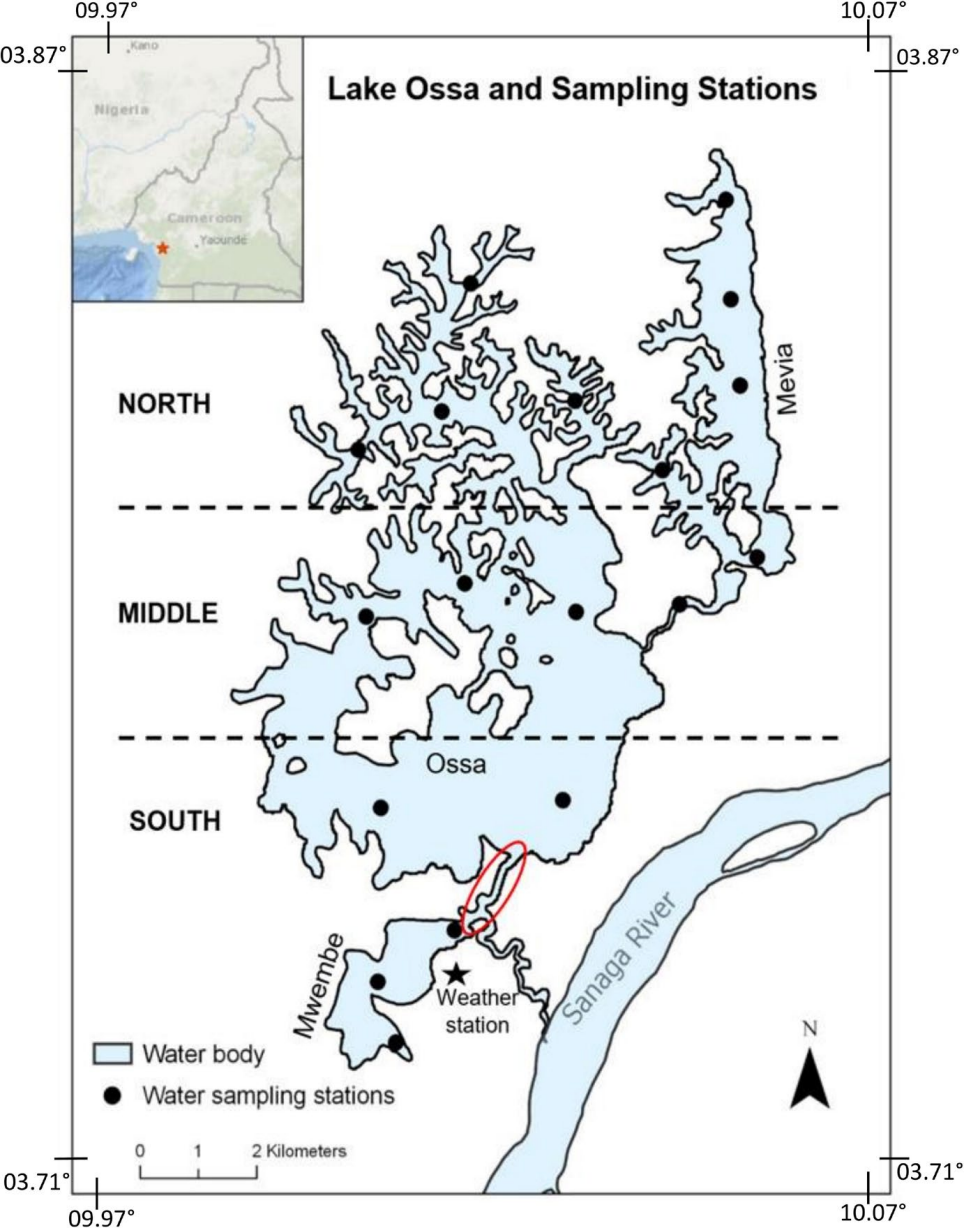
Map of Lake Ossa showing the 18 sampling stations, the weather station location and the arbitrary zones of the lake. The red circle indicates the outlet

The catchment area of the Lake Ossa complex is about 245,000 ha and extends mostly on the northern part, drained by a network of small interconnected near‐perennial streams. The southwestern part of the watershed is very narrow with a steep slope. The lake itself is a lacustrine complex consisting of three lakes (Figure 1). Lake Mévia in the north of the lake complex is 700 ha; Ossa has the largest water surface area of the lakes with 37,000 ha, and Mwembé, the smallest (300 ha), is located at the most southern part of the lacustrine complex. The Lake Ossa complex is shallow with a maximum depth of about seven meters during the wet season (Giresse et al., 2005). The greatest width of the lake is approximately 7 km.

Lake Ossa is adjacent and connected to the Sanaga River through a 3 km meandering and relatively deep channel (5–9 m). Hence, the seasonal incursion of Sanaga water (brownish and full of sediment) into the lake appears to influence its watercolor and chemistry. The water transparency of the lake is not homogeneous across its surface. The banks are mostly covered with weeds dominated by antelope grass (*Echinochloa pyramidalis*) and in some places with shrubs and rattan (*Calamus* sp.). Some of the shorelines have a discontinuity of sandbanks that are the preferred sites for the reproduction of the African Softshell Turtle (*Trionyx triunguis*). In the middle of the rainy season, a large proportion of the littoral forest becomes flooded, providing fresh plant food and refuge for the African manatee and other aquatic species. The Lake Ossa complex lies on a yellow ferralitic bed formed during the Paleocene. The soil is abundant with iron‐rich shales and black marlstones. Successive clay and sand cover shale and black marlstone layers (Giresse et al., 2005).

About 17,000 human inhabitants live around the lake (BUCREP, 2010), and the majority of them depend directly or indirectly on the lake's resources to survive. Over 300 fishers are active on the lake, especially during the low‐water season (Takoukam Kamla, 2012). The local community utilizes the lands around the lake for agricultural purposes. Additionally, there is a large agro‐industry established on the southwest portion of the lake, which has rubber and palm tree plantations covering an area as large as the lake itself.

### 2.2 Data collection

#### 2.2.1 Water chemistry

A total of 18 sampling stations were established, which were distributed evenly across the lake (Figure 1). Surface water sampling was conducted at each station monthly from May through August 2016 and consisted of measuring the transparency (via Secchi disk; see Caffrey et al., 2007; Canfield et al., 1985) directly and total nitrogen (TN), total phosphorous (TP), and total chlorophyll (TChl) concentrations following the “LAKEWATCH” standard operation procedures (Canfield et al., 2002; Hoyer et al., 2012). Chlorophyll was filtered immediately in the field from 300 ml of the lake surface water using a handheld water pump and a Merck Millipore filter paper. Another volume (50 ml) of surface water was collected for TP and TN concentration measurements. The chlorophyll filters and the 50 ml water samples (TN and TP) were frozen (−5°C) until shipped to the Florida LAKEWATCH Laboratory of the University of Florida for analysis. The depth and transparency data of the lake have been monitored in the course of the ongoing Manatee Monitoring Program (MMP) implemented by the African Marine Mammal Conservation Organization (AMMCO). AMMCO established five permanent sampling stations (Figure 1) beginning in January 2016 and has been recording transparency (Secchi depth), water depth, manatee presence, and other biophysical parameters monthly for four to five consecutive days. These data were included in this study to assess the seasonal variation of these variables.

#### 2.2.2 Rainfall

A weather station (Vantage Pro by Davis Instruments) was installed at the AMMCO office in the south of Lake Ossa (Figure 1). The device was installed in January 2016 and collected daily rainfall and uploaded to a cloud account from which monthly averages for rainfall were derived.

#### 2.2.3 Bathymetry and submerged aquatic vegetation (SAV)

Hydroacoustic and GPS data were recorded simultaneously every two to three meters during the survey using the Lowrance Sonar (Lowrance HDS 9 Gen 3) with built‐in GPS capability. The single‐beam, 200 kHz transducer with a 20° beam angle of the Lowrance sonar was mounted on the transom of a small boat that followed predefined‐parallel transects relatively spaced at 100‐m intervals. The boat speed was maintained at approximately 7 km/h. The principle of the translation of acoustic signals to the depth and biovolume data is described in detail in Valley et al. (2015). The biovolume refers to the average percentage of the water column taken up by vegetation. The lake was surveyed from 4 to 10 September 2016, during the highest water season of the year. The raw data (in SL2 format) were downloaded from the Lowrance device and uploaded to the BioBase server for data processing. BioBase is a cloud‐based software platform that generates GIS data layers of depth, biovolume, and bottom hardness from an acoustic and GPS signal (Valley, 2016; Valley et al., 2015). The processed data were delivered as an interpolated grid point. The data accuracy of the SAV data was accessed by ground‐truthing at 50 randomly selected points including 25 with and 25 without vegetation as detected by the Lowrance sonar.

### 2.3 Data analyses

R Studio version 1.1.153 (Team, 2015) and ArcGIS Pro 2.1.3 software (ESRI, 2018) were used for all statistical and geoprocessing analyses.

#### 2.3.1 Water chemistry and rainfall

The monthly values of the water chemistry variables (Secchi depth, TP, TN, TChl) were averaged by the three lake zones delineated perpendicularly to the water flow in the lake (south, middle, and north as illustrated in Figure 1) and for the area as a whole (Table 1). Extreme outliers (greater than 3 standard deviations) were discarded. The average TN/TP ratio was computed to determine whether nitrogen or phosphorus is a limiting factor to algae growth. A simple linear regression model was assessed between TN and TChl, TP and TChl, Secchi depth and TChl, and Secchi depth and distance to the water station at the outlet of the lake. These variable values were also compared with those collected in May 1985 by Wirrmann (1992) in the same lake to assess the dynamics of the lake trophic state.

**TABLE 1:** Surface water chemistry; TP = total phosphorus, TN = total nitrogen, TChl = total chlorophyll concentrations, and SD = Secchi depth

Parameter	South (outlet)	Middle	North	Combined (current)	Combined (historical)
TN (μg/L)	398.9 (110–680)	329.4 (70–930)	327.5 (210–550)	348.9 (70–930)	157
TP (μg/L)	40.6 (8–209)	21.2 (7–46)	25.1 (7–150)	28.2 (7–209)	12.9
TN/TP	15.9 (2.4–42)	15.9 (7.8–34.3)	23.4 (3–50)	19.3 (2.4–50)	12.17
TChl (μg/L)	29.9 (10–53)	17.4 (9–28)	15.2 (9–27)	19.8 (8–53)	8.4
SD (m)	0.8 (0.4–1.2)	1.1 (0.8–1.8)	1.5 (0.8–2.8)	1.2 (0.4–2.8)	NA
Trophic state				Eutrophic	Mesotrophic

Note

Monthly values for all 18 stations sampled from May through August 2016 were averaged by zone (south, middle, and north) of the Lake Ossa and combined for the entire lake. Historical values were measured in May 1985 by Wirrmann (1992). Values in the parentheses represent monthly minimum and maximum. Trophic state categorizations were based on Carlson (1977).

The Secchi depth and water depth data from the MMP, as well as the rainfall data recorded in 2016 and 2017, were averaged by month. The maximum depth of plant colonization (MDC) values at each of the water stations was predicted based on the Lambert–Beer equation (1):

(1) $$\text{MDC}=\frac{‐\ln\left(\frac{I_{z}}{I_{0}}\right)}{K}$$

where *I_z_* is the light intensity at depth *z*, *I*_0_ is the light intensity at the surface, (*I_z_*/*I*_0_) is the percentage of light at MDC of the submerged aquatic plant of interest. Chambers and Kalff (1985) assessed the maximum depth of colonization (MDC) for charophytes and angiosperms and estimated that on average, they occur at 11% and 21% of the surface incident irradiance, respectively. Because angiosperm plants dominate Lake Ossa, a maximum target depth of SAV colonization was set at 20% (i.e., *I_z_*/*I*_0_ = 0.2). *K* is the light attenuation coefficient usually measured by a light meter. A lightmeter was not available for use in this study, so we used an alternative estimator based on Secchi depth (SD) values following the equation by Poole and Atkins (1929) which is applied worldwide:

(2) $$K=\frac{1.7}{\text{SD}}$$

The predicted MDC values along with the Secchi depth and depth were plotted on the same date scale graph to graphically visualize how likely SAV will occur over the year.

#### 2.3.2 Spatial analysis and prediction

The bathymetry grid point data were plotted on ArcGIS and converted into a 16‐m cell size raster layer using the conversion tool of the software. Then, the cells were classified by equal interval depth ranges of one meter. The light attenuation coefficient was used to predict the percentage of surface irradiance (*I*_0_/*I_z_*) at the bottom (of depth *Z*) of each of the raster cell based on the Lambert–Beer equation

(3) $$\frac{I_{z}}{I_{0}}=e^{‐KZ}$$

where *I_z_* and *I*_0_ are the light intensities at the bottom (depth *z*) and the water surface, respectively. Given the *K* proxy in Equation (2), the percent irradiance at a bottom *z* (Equation 3) can be rewritten as follows:

(4) $$\frac{I_{z}}{I_{0}}=e^{‐1.7Z/\text{SD}}$$

Thus, the percentage irradiance depends only on the depth *z* (bathymetry data) and the SD values. Because there was a strong relationship between SD values (*Y*) at each sampling station and the Euclidean distance (*X*) of the station to the outlet of the lake, the linear regression (Equation 5) was used to predict the SD value at each raster cells using the raster calculator. The Euclidean distance is the straight‐line distance between two points.

(5) Y=0.073X+0.69

The straight linear distance (*X*) of each raster cell to a source cell situated at the outlet of the lake was calculated using the Euclidean Distance function of the Spatial Analysis tool of ArcGIS. Then, Equation (5) was applied to the Euclidean Distance raster layer to generate a predicted SD layer using the Raster Calculator function of the Spatial Analysis Tool. The predicted SD and the bathymetry raster layers were used to generate the predicted percent irradiance at bottom (*I_z_*/*I*_0_) layer by applying Equation (4) using the Raster Calculator function. Finally, the latter layer was classified as follows:

- Class 1: Raster cells predicted to receive <15% of surface irradiance at bottom and therefore assumed to bear no SAV.
- Class 2: Raster cells predicted to receive between 15% and <25% of surface irradiance at bottom and expected to bear moderate SAV if only light availability was limiting.
- Class 3: Raster cells predicted to receive ≥25% of surface irradiance at bottom and therefore expected to bear abundant SAV if only light availability was limiting.

The observed SAV grid point data collected through the Lowrance Sonar was processed using the same procedure as with the bathymetry data described above. Cells with biovolume <10% were classified as noise or insignificant (Class 1). Cells with biovolume between 10% and <25% were classified as moderate SAV (Class 2), and abundant SAV (Class 3) were cells with biovolume ≥25%.

#### 2.3.3 Accuracy assessment

To assess the correlation between our predictive model and the observed SAV distribution, a confusion matrix (Boughorbel et al., 2017) was constructed from a random subsample representing 20% (35,708 cells) of the total cells across the lake. For each of the subsample cells, corresponding values from both the predicted SAV layer (Figure 4c) and the observed SAV (Figure 4d) as detected through the Lowrance Sonar were extracted using the Extract by Point function of the Spatial Analysis Tool of ArcGIS. The extracted data were then transformed to a binomial data where class 1 of both layers was assigned to “absence,” and classes 2 and 3 of both layers were assigned to “presence.”

The number of true‐ and false‐positive cells, as well as the number of true‐ and false‐negative cells, were counted. True positive (Tp) and true negative (Tn) are cells for which both the observed and the predicted values correspond to “presence” and “absence,” respectively. False positive (Fp) are cells that observed SAV value is “absence” and predicted value is “presence.” False negative (Fn) are cells that observed SAV value is “presence” and predicted value is “absence” (Table 2). The confusing matrix performance metrics (Fawcett, 2006; Matthews, 1975) were calculated including the error rate, accuracy, sensitivity, specificity, precision, false‐positive rate, and Matthews correlation coefficient (Table 3).

**TABLE 2:** Confusion matrix parameter values

		Predicted		
		Positive	Negative	
Observed	Positive	Tp = 184	Fn = 450	Op = 634
	Negative	Fp = 1133	Tn = 33,941	On = 35,074
		Pp = 1317	Pn = 34,391	T = 35,708

Abbreviations: Fn, false negative; Fp, false positive; On, total negative observed; Op, total positive observed; Pn, total negative predicted; Pp, total positive predicted; T, the total number of cells assessed; Tn, true negative; Tp, true positive.

**TABLE 3:** Confusion matrix performance parameter values, formula, and abbreviations used for the submerged aquatic vegetation predictive model in Lake Ossa

Performance metric	Abbreviation	Formula	Value
Error rate	ERR	$\frac{\text{Tp}+\text{Fn}}{T}$	4%
Accuracy	ACC	$\frac{\text{Tp}+\text{Tn}}{T}$	96%
Sensitivity (true‐positive rate)	SN	$\frac{\text{Tp}}{\text{Op}}$	29%
Specificity (true‐negative rate)	SP	$\frac{\text{Tn}}{\text{On}}$	97%
Precision (positive predictive value)	PREC	$\frac{\text{Tp}}{\text{Pp}}$	14%
False‐positive rate	FPR	$\frac{\text{Fp}}{\text{On}}$	3%
Matthews correlation coefficient	MCC	$\frac{\text{Tp}*\text{Tn}‐\text{Fp}*\text{Fn}}{\sqrt{(\text{Pp}}*\text{Pn}*\text{Op}*\text{On})}$	0.18

Abbreviations: Fn, false negative; Fp, false positive; On, total negative observed; Op, total positive observed; Pn, total negative predicted; Pp, total positive predicted; T, the total number of cells; Tn, true negative; Tp, true positive.

## 3 RESULTS

### 3.1 Water chemistry and rainfall

All water chemistry variables indicated a level of gradient from the south (around the outlet of the lake) toward the north (farthest areas from the outlet). The average values of TN, TP, and TChl were highest in the south and were lower in the middle and the northern zones of the lake. The overall average values (TN, TP, and TChl) for the lake were more than double the values recorded by Wirrmann (1992) in May 1985 (Table 1). The average Secchi depth and the ratio of TN to TP concentrations (TN/TP) were lower in the south and middle and higher in the north zone of the lake (Table 1). The whole lake TN/TP ratio grand mean was greater than 17 (19.3), suggesting that Lake Ossa chlorophyll growth is most likely phosphorus limited at least from May through September (corresponding to the sampling period of this study; Sakamoto, 1966; Smith, 1982).

**FIGURE 2:**
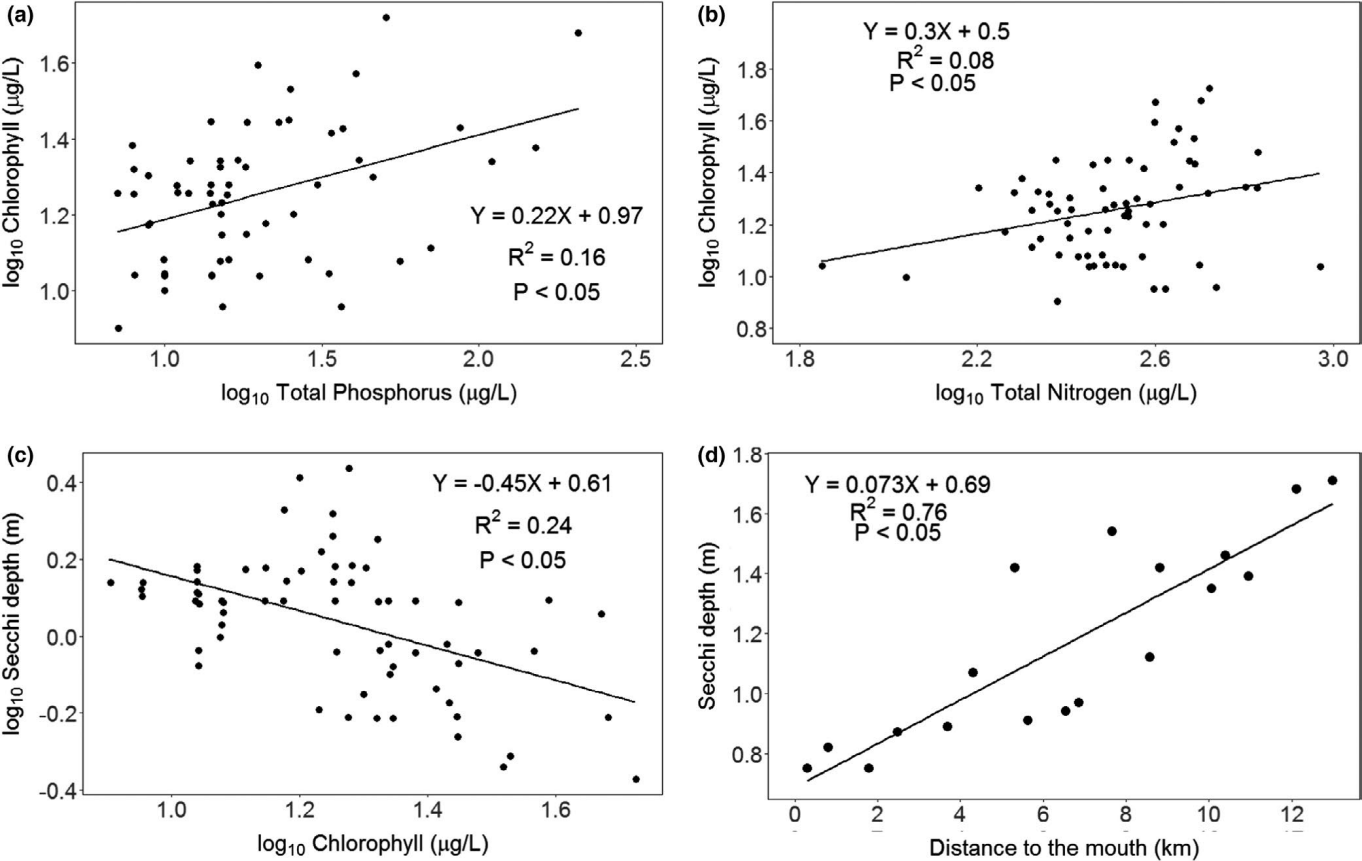
Effects of phosphorous and nitrogen on chlorophyll concentrations (a & b) and chlorophyll and distance to the outlet on Secchi depth (c & d) for all 18 stations sampled in Lake Ossa between May and August 2016. (a) Relationships between monthly TP (µg/L) and monthly TChl (µg/L) concentrations; (b) between monthly TN (µg/L) and total chlorophyll concentrations; (c) relationships between monthly Secchi depth (m) and monthly Tchl (µg/L) concentrations; (d) between station average Secchi depth (m) and distance to the outlet of each station (km)

There were significant but weak positive linear relationships between TP and TChl concentrations (Figure 2a), between TN and TChl concentrations (Figure 2b) with phosphorus and nitrogen accounting, respectively, for 16% and 8% of the variance in TChl concentrations of Lake Ossa. There was also a significant, but weak negative linear relationship between Secchi depth and TChl concentration with the later accounting for 24% of the variance in the observed Secchi depth (Figure 2c). The Euclidean distance of the sampling points to the outlet of the lake surprisingly showed a significant relationship with the average Secchi depth at each sampling point (Figure 2d). The distance of the sampling point to the outlet accounted for 76% of the variance in the observed Secchi depth (*r* = .76, *p* < .05). Further assessment, using multivariate linear regression analyses with Secchi depth as the dependent variable and TChl concentration and distance of the sampling point to the lake outlet as the explanatory variables showed that only the distance of the sampling was significant (*p* = .001). After accounting for the distance of the sampling point to the outlet, TChl concentration accounted for only another 2.8% of the variance in Secchi depth (*R* ^2^ = 0.78, *p* < .05).

**FIGURE 3:**
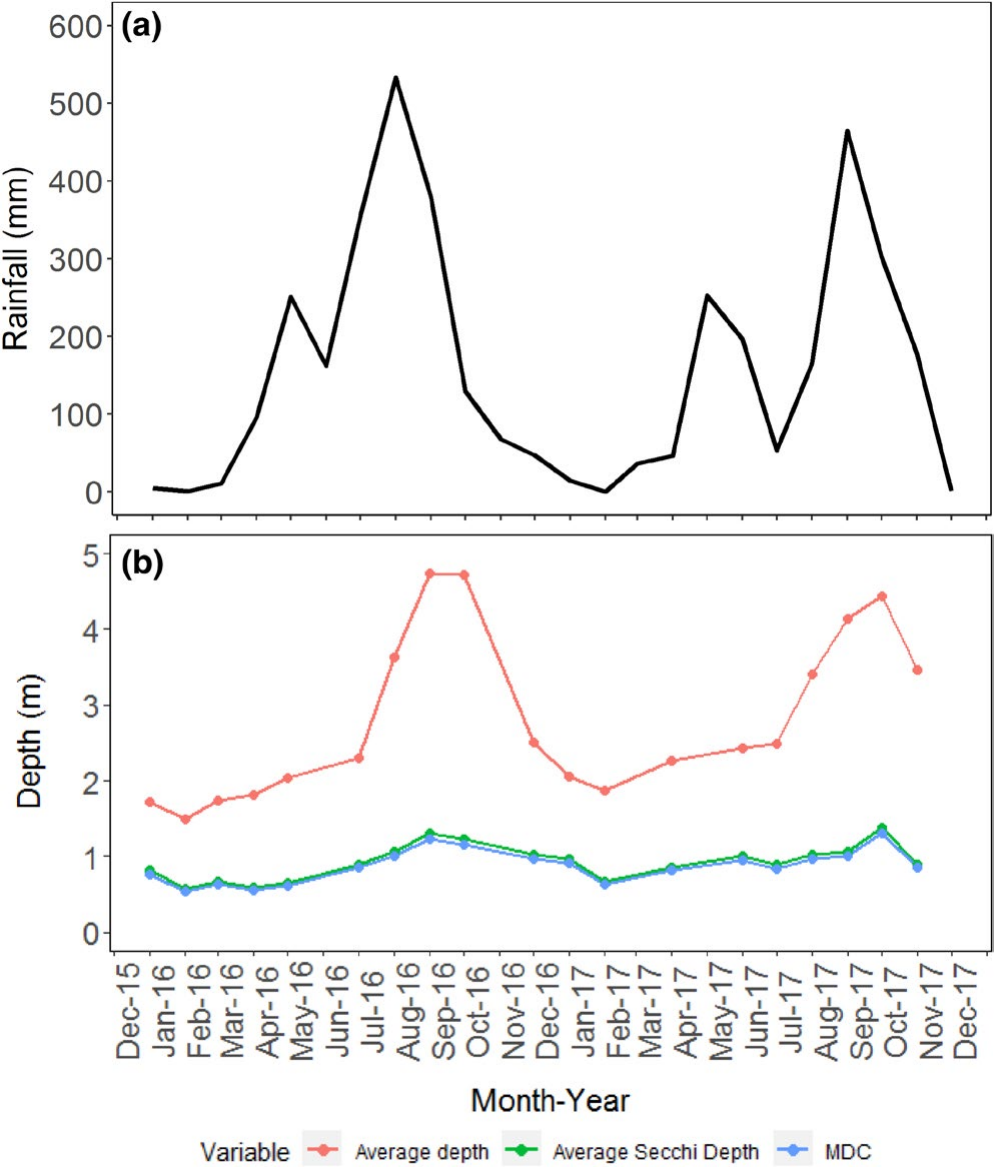
Monthly variations of rainfall, depth, Secchi depth, and MDC. Comparison of monthly rainfall: (a) with monthly averages of water depth, Secchi depth, and a maximum depth of plant colonization (MDC), and (b) measured in the five permanent sampling stations in Lake Ossa in 2016 and 2017

The monthly mean rainfall recorded in Lake Ossa over the two years (2016 and 2017) identified two seasons (Figure 3a): the rainy season from April to October with a short peak (about 250 mm) in May and the highest peak in August or September (about 500 mm) and then a dry season from November to March. The monthly variation of the average water level in the lake follows approximately the same variation pattern as the rainfall with a delayed peak a month behind the main rainfall peak (Figure 3b). The water level increased by about 2.5 m from the dry to the rainy season.

The monthly mean Secchi depth measured in the five permanent stations during the two years and the predicted MDC (using Equation 1) follow the same variation pattern with the monthly mean water depth (Figure 3b). Monthly Secchi depth varied from 0.57 m to 1.39 m and MDC from 0.54 m to 1.31 m. The MDC yearly curve (Figure 3b) appears to be way above the lake bottom throughout the year. This gap between MDC and the bottom is larger (up to 3 m) during the high‐water season and smaller during the low‐water season (at least 1 m).

### 3.2 Spatial analysis and prediction

**FIGURE 4:**
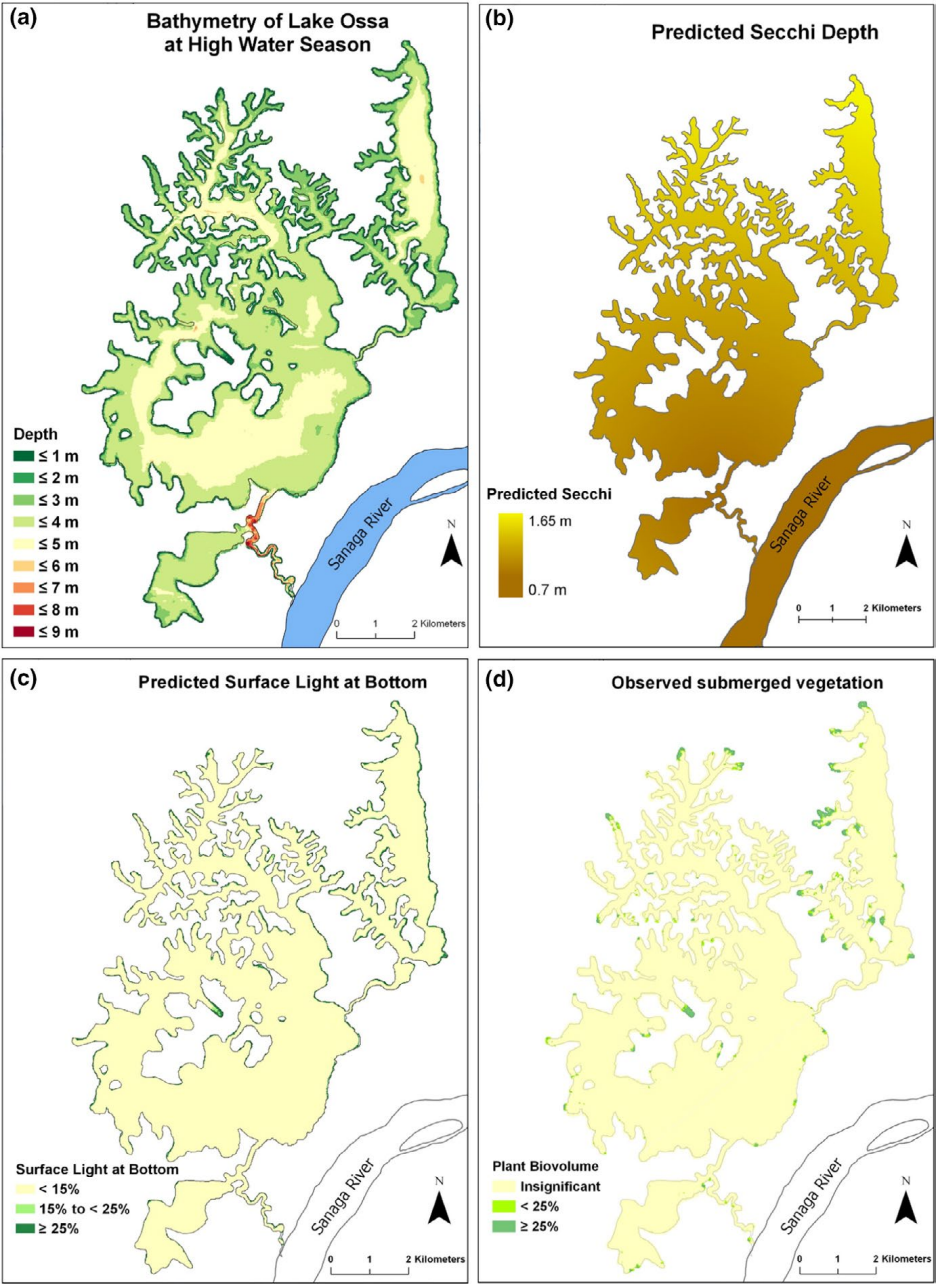
Mapped measurements and predicted values at Lake Ossa, Cameroon. (a) Bathymetry map of Lake Ossa showing the different depth gradients. The bathymetric points were recorded in September 2016 during the wet season. (b) Prediction of Secchi depth (visibility) throughout Lake Ossa, based on the established linear relationship between the Euclidean distance of a sampling point for the outlet of the lake and Secchi depth at that point. Yellow areas comprise higher water visibility, whereas brown indicates lower water visibility. (c) Prediction of percent of surface light available at the bottom throughout the Lake Ossa. The areas in yellow are those where no SAV is expected, while the areas in green are those where SAV presence is expected. (d) Map of the distribution of the SAV in Lake Ossa measured using the Lowrance HDS 9 Gen 3. Values were measured in September 2016. The areas in yellow are those where no SAV is expected, while the areas in green are those where SAV presence is expected

The bathymetric map showed that Lake Ossa is a shallow water system with depths less than 5 m within the lake basin and up to 9 m in the channel connecting the lake to the Sanaga River (Figure 4a). An average increase of 2.5 m of the water depth from the low dry to the high rainy season implies that water depth in the entire lake basin is less than 2.5 m during the dry or low‐water season, with most of the lake having a depth under 1.5 m. The water surface areas during the high‐water season were estimated to be at least 4900 ha and the volume to be at least 161 million m^3^.

The spatial distribution map of the Secchi depth (Figure 4b) was predicted based on the linear regression model (Equation 5) explaining the relationship between the Secchi depth and the distance of the sampling point to the outlet of the lake (Figure 2d) because it explained about 76% of the variation in the Secchi depth values. The predicted Secchi depth map showed a strong, increasing gradient moving from the south to the north of the lake with the value varying from 0.7 m to 1.65 m.

Mean *K* estimated values for each of the 18 water sampling sites during the four sampling months varied from 1.04 to 2.74 m^−1^ with a grand mean of 1.71 m^−1^. The monthly mean *K* estimated values for the five permanent sampling sites together for two years (2016 and 2017) varied from 1.2 to 3.0 m^−1^ with an annual mean of 2.1 and 1.8 m^−1^ for the years 2016 and 2017, respectively. The monthly direct normal irradiance recorded in Lake Ossa in 2016 varied from 42,473 W m^−2^ in August to 112,918 W m^−2^ in January (ParisTech, 2018).

The model predicted that only about 3.7% of the lake surface bottom received more than 15% of the surface light irradiance and therefore was more likely to sustain submerged vegetation. The spatial model predicted that the SAV is distributed only on the edges of the lake and more abundant toward the middle and in the north zone of the lake (Figure 4c). Similarly, the observed SAV data indicated that only about 2% of the bottom is occupied with SAV which has a similar marginal distribution pattern of a predicted distribution (Figure 4d). Both the predicted and the observed values indicated that SAV is almost entirely absent in Lake Ossa.

The confusion matrix analysis revealed that the prediction model performed strongly (Tables 2 and 3). The error and the false‐positive rates were only 4% and 3%, respectively, while the accuracy and the specificity were 96% and 97%, respectively. The sensitivity (29%) and the precision (14%) of the model were both moderate.

## 4 DISCUSSION

We set out to understand African manatee habitat suitability in Lake Ossa during the low‐ and the high‐water seasons. Lake Ossa trophic states parameters (TN, TP, and TChl) doubled in three decades, moving from a mesotrophic to a eutrophic state based on Carlson's trophic state classification (Carlson, 1977), which is the most commonly used trophic state classification index (Devi Prasad & Siddaraju, 2012). Thus, the lake system went from a moderate to a high level of biological productivity. However, the lake appears to sustain only a limited level of fish and submerged plant productivity because of poor water clarity that limits the amount of light available for phytoplankton growth, which in turn reduces fish biomass and diversity (Chambers & Kalff, 1985). Increased nutrient concentrations in the lake seem to have contributed to the proliferation of an invasive aquatic fern (*Salvinia molesta*) that was first observed in 2016 and 2017.

The decreasing nutrient gradient (TP and TN) moving from the outlet of the lake (in the south) to the north indicates that the flow of the Sanaga River is the main source of nutrients for the lake. The Sanaga River is the major river of Cameroon, measuring 918 km long with a drainage basin close to 140,000 km^2^, representing about 30% of the national territory (Van der Waarde, 2007). Thus, the Lake Ossa drainage basin, which is only 245 km^2^ wide (Wirrmann & Elouga, 1998), may only contribute marginally to the nutrient load when compared to the large Sanaga catchment area.

Three main anthropogenic factors could be responsible for the nutrient load increase in the Sanaga River: forest clearing, farming, and urbanization. Cameroon loses approximately 220 kha of forest every year (Food and Agriculture Organization of the United Nations (FAO), 2010). The forest clearing in Cameroon exposes soils and accelerates erosion, sediment loading, and organic matter deposit in the rivers, leading to increased nutrient loading. This loss is mostly driven by clearing for agriculture and urbanization (Epule, 2014). Fertilizers used in agriculture are rich in phosphorous and nitrogen and likely add to the nutrient load increase.

Chlorophyll concentrations and Secchi depth values followed an opposite gradient in Lake Ossa. As chlorophyll decreased from the south (near the outlet) to the north, Secchi depth increased (Table 1). This observation supports two hypothetical scenarios: (a) The increased concentration of chlorophyll generated in Lake Ossa is responsible for the decrease in water clarity; and (b) the bulk of Lake Ossa chlorophyll arrives with the Sanaga flow, and therefore, water clarity is influenced mainly by suspended sediment loading from the Sanaga River rather than the increased chlorophyll concentration.

Lake Ossa has two seasonal flows (Takoukam Kamla, 2012). During the high‐water season, inflow from the Sanaga River introduces suspended sediments and chlorophyll to the lake. These suspended particles settle out of solution as the water flows further into the lake, increasing water clarity. Water sampling in this study was done during the high‐water season. When assessed independently, the distance of the sample site from the Sanaga inflow accounted for 76% of the variation in Secchi depth, while chlorophyll concentrations accounted for only 24% (Figure 2c,d). Multivariate analysis further demonstrated that chlorophyll concentration did not contribute significantly to water clarity. Therefore, during the high‐water season, scenario 2 is likely dominant.

During the low‐water season, the lake water flows back into the Sanaga River. The clarity during that season is therefore expected to increase as there are less suspended sediments flowing from the river into the lake. However, our results show that clarity instead decreased during the low‐water season (Figure 3b). The main explanation for this decrease lies in the fact that during the dry season, the low‐water level (less than 2.5 m) exposes the water bottom to the strong forces of the dominant Harmattan winds that may displace sediment from the bottom. Also, during periods of the high‐water season, when the direct water input from the rain dominates the water input from the Sanaga River, it may contribute to the dilution of the sediments, and thus increasing the water clarity of lake. Unfortunately, we did not measure the chlorophyll concentrations in the lake during the low‐water season. However, given the lower water clarity at that season, we suspect that the chlorophyll concentration should be lower compared with the high‐water season and that there would be no north–south gradient as observed during the rainy season. Nonetheless, the high‐water clarity will lead to increased chlorophyll concentration in areas of the lake basin that are less exposed to the winds in Lake Mevia (Figure 1), which is surrounded by hills and high trees. Thus, scenario 1 would likely occur in those areas sheltered from the direct wind and neither scenario 1 nor 2 would occur in areas readily exposed to the wind.

**FIGURE 5:**
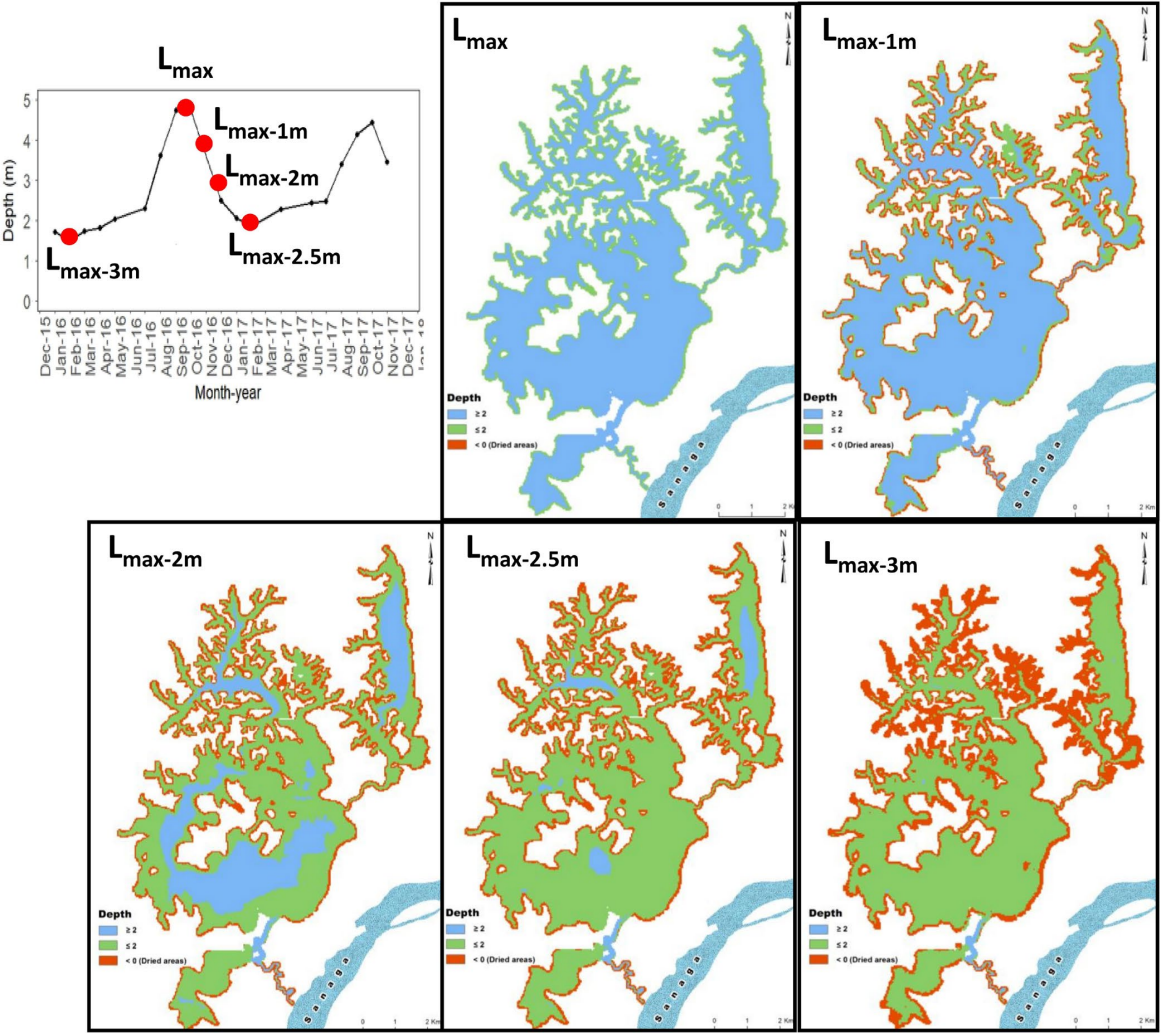
Prediction of manatee habitat suitability based on the seasonal water variation in Lake Ossa. The spatiotemporal modeling of the bathymetry was based on the average monthly water depth data recorded at one point of Lake Ossa between December 2015 and December 2017. The initial bathymetry map was conducted when water level was at highest (Lmax, top second frame) during the raining season; then, simulations were performed for series of decrease in water level by 1, 2, 2.5, and 3 m consecutively. Areas in blue are those with a depth of at least 2 m and assumed to be suitable for manatees; areas in green are those less than 2 m and less suitable for the manatees; areas in red are those that become dried as water level recedes

Lake Ossa, like the rest of the lakes in Cameroon, does not have existing bathymetric data. Therefore, we conducted the first bathymetry measurements of Lake Ossa. The bathymetry and SAV mapping of lakes has become easier with the advent of hydroacoustic and global positioning systems (Abd Ellah, 2016; Valley et al., 2005). Local fishers believed that there are many “manatee holes” (deep depressions) in the lake in which manatees take shelter to avoid detection (Takoukam Kamla, 2012). Our bathymetric map does not seem to agree with the fishers' beliefs. Lake Ossa's main basin bottom relief seems very regular, flat, and shallow. The only relatively deep part of the lake is located along the creek connecting the lake to the Sanaga River (Figure 4a). The creek is almost two times deeper than the main lake. Simulation on our bathymetric map using a 2.5 m water depth decrease from the high‐ to the low‐water season indicates that during the low‐water period, 38% and 94% of the lake area has a depth less than 1 m and 2 m, respectively (Figure 5). Generally, manatee suitable habitat includes areas of at least 2 m deep (Reep & Bonde, 2006; USFWS, 1999) and only 6% of the lake bottom meets that criteria; thus, based only on water depth, Lake Ossa may not be an ideal refuge for the African manatees during the low‐water season as the lower reaches of the Sanaga River provide greater depths at that time of the year.

The surface light at the bottom model (assuming a light attenuation coefficient *k* = 1.7/SD) predicted that only 4% of Lake Ossa received sufficient light at the bottom (>15% of surface irradiance) through at least four months during this study. Because the data collection was conducted only during the rainy season, which is the time of year with the lowest direct normal irradiance and lowest water depth, we might expect a slightly different result during the dry/low‐water seasons despite the offset of the decreased Secchi depth during that season (Figure 3). Our model prediction did not change significantly when using different *K* values (*K* = 1.6/SD, *K* = 1.8/SD, *K* = 2.0/SD), probably because of the low values obtained from the Secchi depth.

Our model prediction of the distribution of SAV in Lake Ossa was in strong agreement with the observed SAV distribution (Figure 4d); both indicating that SAV is absent across almost the entire lake bottom (96% and 98%, respectively). The confusion matrix analysis (Tables 2 and 3) further confirmed the high performance of our predictive model as indicated by the low error rate (4%) and high accuracy (96%). Our predictive model relied on only two main variables including Secchi depth spatial distribution and bathymetry. There, we demonstrated that the spatial distribution of Secchi depth in the lake could be extrapolated from a single point at the outlet using Equation (5). The bathymetry map can be updated by applying the change in water level at one point to the entire water surface depth data. Therefore, we demonstrated that we could monitor the distribution of SAV in Lake Ossa by monitoring the Secchi depth value and the water depth from a single permanent sampling point, placed ideally near the outlet of the lake. However, our model needs to be improved by including data from the low‐water season and expanding data collection for several years to account for seasonal and annual variability.

The bathymetry data and the SAV distribution of Lake Ossa suggest that very few areas of the lake remain suitable for the species during the low‐water season. This observation further supports the hypothesis that manatees migrate out of the lake during the low‐water season and return during the rising‐water season when the emergent vegetation becomes flooded. Such a migratory pattern in relation to high and low water has been documented in other African manatee populations in many countries throughout Africa such as Sierra Leone, Gambia, and Ivory Coast (Akoi, 2004; Powell, 1996; Reeves et al., 1988) as well as in other manatee species such as the Amazonian manatee (Arraut et al., 2010; Deutsch et al., 2003; Marmontel et al., 2002) and the Antillean manatee (Colmenero‐Rolón, 1986; Reynolds & Odell, 1991; Reynolds et al., 1995).

The low quality of manatee habitat in Lake Ossa during the low‐water season demonstrated by the current study contrasts with the higher frequency of manatee sightings during the same season as documented by the MMP (unpublished data). Yet, the higher manatee sighting frequency during the low‐water season does not necessarily imply an increase in abundance. The more plausible explanation is that manatees in Lake Ossa during the low‐water season are less cryptic, and therefore more easily detectable as their habitat is restricted to small, relatively shallow areas. During the higher water season, sightings are rare as access by biologists is limited when the manatees migrate into the flooded forest and swamp. Thus, using survey approaches that will estimate abundance with detection error will be crucial to determine the seasonal dynamic of the manatee population in Lake Ossa.

Like many developed countries, Cameroon has strong environmental laws. However, the level of enforcement of the law is apparently very weak in Cameroon. It is important to reinforce manatee patrols and fishery management in Lake Ossa, especially during the dry season as the individual manatees that did not migrate out of the lake system may be more vulnerable to hunting and bycatch. Because food accessibility is limited, manatees may take the risk of grazing in very shallow areas where they can easily be detected and trapped by potential hunters. The information generated through this study will be crucial for the future elaboration of the first management plan of the lake. The eutrophication of Lake Ossa, and subsequent lack of submerged vegetation, is possibly common to other aquatic habitats in Cameroon, which means African manatees may not have as many high‐quality refuges as we thought and may be migrating to Lake Tissongo further downstream Sanaga River where they may have access to deeper and less eutrophic waters. Genetic analysis based on microsatellites indicated that manatees in Lake Ossa, Sanaga, and Tissongo are the same population, and therefore, they migrate between these locations (unpublish data). Telemetric monitoring of some individuals could help determine the seasonal and spatial patterns of the migration. Law enforcement, population monitoring, and habitat restoration are all necessary if we hope to save this species from extinction.

## CONFLICT OF INTEREST

The authors declare no conflicts of interest.

## AUTHOR CONTRIBUTIONS

**Aristide K. Takoukam:** Conceptualization (lead); data curation (lead); formal analysis (lead); funding acquisition (lead); investigation (lead); methodology (lead); resources (lead); visualization (lead); writing‐original draft (lead); writing‐review & editing (supporting). **Dylan G. E. Gomes:** Data curation (supporting); visualization (supporting); writing‐original draft (supporting); writing‐review & editing (lead). **Mark V. Hoyer:** Conceptualization (supporting); methodology (supporting); resources (supporting); validation (lead); writing‐review & editing (supporting). **Lucy W. Keith‐Diagne:** Supervision (supporting); writing‐review & editing (supporting). **Robert K. Bonde:** Funding acquisition (supporting); methodology (supporting); project administration (lead); supervision (lead); writing‐review & editing (supporting). **Ruth Francis‐Floyd:** Funding acquisition (supporting); methodology (supporting); project administration (lead); supervision (lead); writing‐review & editing (supporting).

### OPEN RESEARCH BADGES

This article has earned an Open Data, for making publicly available the digitally‐shareable data necessary to reproduce the reported results. The data is available at http://doi.org/10.5281/zenodo.4296940.

## Data Availability

All data and code are available as a Zenodo dataset at http://doi.org/10.5281/zenodo.4296940.
